# Patient Involvement in Education of Nutrition and Dietetics Students: A Systematic Review

**DOI:** 10.3390/nu11112798

**Published:** 2019-11-16

**Authors:** Judi Porter, Nicole Kellow, Amanda Anderson, Andrea Bryce, Janeane Dart, Claire Palermo, Evelyn Volders, Simone Gibson

**Affiliations:** 1 Allied Health Clinical Research Office, Eastern Health, Box Hill, Victoria 3128 Australia; 2 Department of Nutrition, Dietetics & Food, Monash University, Notting Hill, Victoria 3168, Australia; nicole.kellow@monash.edu (N.K.); amanda.anderson@monash.edu (A.A.); andrea.bryce@monash.edu (A.B.); janeane.dart@monash.edu (J.D.); claire.palermo@monash.edu (C.P.); evelyn.volders@monash.edu (E.V.); simone.gibson@monash.edu (S.G.)

**Keywords:** patient involvement, nutrition and dietetics, education, systematic review

## Abstract

A client-centred approach sits at the core of modern healthcare. Exploration of the patients’ role within the education of nutrition and dietetic students has not previously been undertaken. This review aimed to synthesise the learning outcomes that result from involvement of patients in nutrition and dietetic student education, and to consider whether these interactions promote patient-centred care. Five electronic databases were searched, supported by hand-searching of references of included studies. Screening of title/abstract and then full text papers was undertaken; key characteristics and outcomes were extracted and synthesised narratively. The likely impact of interventions was evaluated using Kirkpatrick’s Hierarchy; study quality was assessed using the Medical Education Research Study Quality Instrument and Critical Appraisal Skills Programme checklist. Of 7436 studies identified through database searching, and one additional study located through hand searching of reference lists, the final library consisted of 13 studies. All studies reported benefits for student learning from patient involvement, while one paper identified patient benefits from student interventions. Patients as recipients of care mostly contributed in a passive role in student education activities. Quality assessment identified methodological limitations in most studies. Patient involvement in the education of dietitians supports skill development and therefore progression to professional practice. Although nutrition and dietetics education has a focus on client-centred care, the translation of these concepts into an interactive student educational experience has been investigated to a limited extent. Collaboration with patients in student education is an area for further development.

## 1. Introduction

There is variability in academic programmes in nutrition and dietetics internationally [1]; however, inherent to each is student progression though performance increments or stages of proficiency. Through education and experience, student progression results in entry-level competence as a dietitian/nutritionist by programme completion. An essential component of this transition to professional practice [2] is the development of effective nutrition counselling skills, usually undertaken through interactions with service users (including patients, clients, and other end users) of nutrition and dietetic services. Service users, hereafter referred to as patients, have valuable contributions to offer beyond their role as recipients of healthcare. Their interactions in an educational capacity have benefits for health students including enhanced skill development, confidence, fostering reflexivity, and other professional attributes [3]. 

Modern healthcare aims to have patient engagement at the centre of its delivery, and opportunities have been explored for integrating patients into the design and evaluation of service delivery [4,5]. However, there are significant gaps in patient-centred care delivery due to individual and system-level limitations [6]. Optimal student training in this area is required to help address this gap. In nutrition and dietetics, the interfaces between professionals, students, and service users were explored in a recent critical synthesis of the literature by Sladdin et al. [7]. Authors identified key themes to establishing patient-centred care in dietetics, including establishing a positive dietitian–patient relationship, and demonstrating effective communication skills and authentic humanistic behaviours. Individualisation of care to patients’ unique needs and wants with shared decision making was central to shifting power to the patient and in facilitating greater involvement in their care [7].

Creating a culture of embedding true patient-centred care into dietetic practice requires education and resources [8], and begins with student training. Within education, this collaboration between patient and student encourages the patient to take on an educator role while the student is a learner and co-educator [9]. As an expert in their condition [10], the patient’s provision of educational experiences for students can lead to powerful and transformative educational experiences [3]. Patients (both authentic and simulated) may contribute to classroom teaching and curriculum development; their contribution has been shown to enhance teaching and learning experiences [11]. The patient may also contribute in their role as a resource for learning and provide encouragement for students [12]. In addition to the healthcare benefits that may rise from the service user–student interaction, patients may benefit individually through communicating and processing their problems [13,14] and feelings of appreciation [14].

The meta-narrative review by Rowland et al. [15] reported that the body of literature surrounding patient involvement in health professionals’ education is complex. This review [15] did not explicitly seek to synthesise studies in nutrition and dietetics. Key concepts that emerged within other health professions included “nothing about me without me”, where patients have rights to shape all aspects of their own care, including education, patients as teachers, real patients as simulated patients, and students learning through involvement in patient care [15]. Authentic (i.e. not tokenistic) representation, therapeutic benefits, and compensation ethics were considered [15]. 

Exploration of the outcomes that arise from patient involvement within nutrition and dietetic student education has not previously been undertaken. This review aimed to synthesise learning outcomes that result from involvement of patients in nutrition and dietetic student education, and to consider whether these interactions promote patient-centred care.

## 2. Materials and Methods 

This review followed the Preferred Reporting Items for Systematic Reviews and Meta-Analyses (PRISMA) guidelines [16] and was prospectively registered with the International Prospective Register of Systematic Reviews (CRD42019124085) [17].

**Figure 1:**
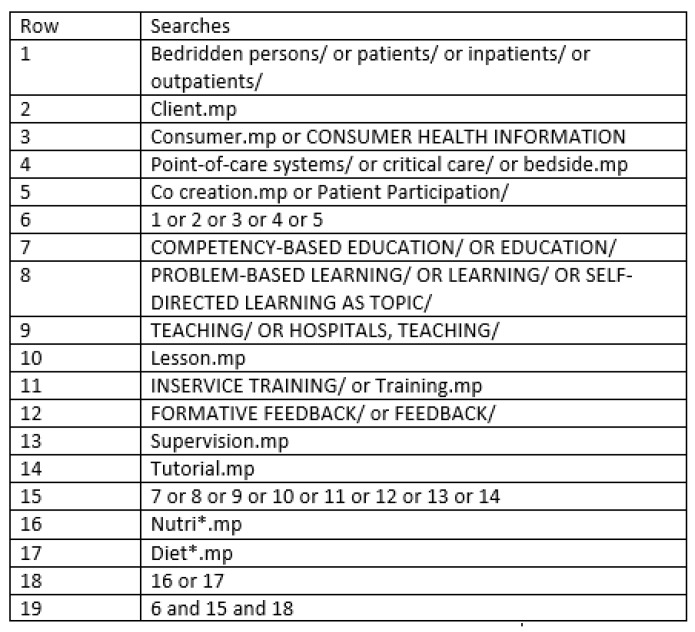
Database search strategy applied in Ovid MEDLINE. * indicates truncation.

Searches were run in five databases: Ovid Medline, CINAHL, PsycINFO, Scopus, and ERIC from database inception to 30 November, 2018. Search terms were determined through refinement of those utilised in the meta-narrative review of Rowland et al. [15]. The full search strategy implemented in MEDLINE is presented in Figure 1. This strategy was then adapted as appropriate for the other databases. There were no limits on outcomes or study design placed on the search strategy, nor were restrictions on setting or language applied. Database searches were imported into Endnote (version 8.2) [18], where duplicate articles were removed. 

The Participant-Intervention-Comparison-Outcomes (PICO) format [19] was used to develop inclusion and exclusion criteria. Studies investigating education of nutrition and dietetics students interacting with patients (or clients or users, including simulated patients) were considered. Broader health system approaches (e.g., public health interventions) and patient care/treatment that did not involve teaching/learning were ineligible. Outcomes of interest were student learning outcomes and patient-related outcomes (including health outcomes). Full papers of original research (qualitative or quantitative) were eligible; conference abstracts, commentaries, and systematic reviews were excluded. Papers that evaluated broader aspects of objective structured clinical examinations but did not report on the specific results of simulated patient interviews, and studies reporting interprofessional patient education where data pertaining to nutrition and dietetics students were not able to be separately extracted were also excluded.

Study selection was undertaken using Covidence [20]. Studies were selected by review of titles and abstracts as well as full text papers against the inclusion/exclusion criteria. Each stage was conducted by authors working independently and in duplicate. Discrepancies were resolved by a third team member. Reviewers did not screen their own authored publications. Reference lists of included publications and key systematic reviews were hand-searched to identify additional studies for inclusion.

Data were extracted into a piloted worksheet detailing key study characteristics (including study location and design and demographic details describing patients and students), student learning outcomes, and patient-related outcomes. One reviewer extracted all data; data extracted from reviewers’ own authored publications were cross-checked by a second author. Analysis was undertaken narratively to synthesise the student learning outcomes and patient-related outcomes.

Methodological quality of quantitative studies was evaluated using the Medical Education Research Study Quality Instrument [21]. This tool is widely used to evaluate medical education research that uses quantitative study designs and yields an overall score of between 4.5 and 18. The Critical Appraisal Skills Programme checklist (CASP) [22] was used to evaluate the quality of studies using qualitative and mixed method studies. Two reviewers (JP and NK) independently assessed each publication, with discrepancies resolved through discussion to reach consensus.

The likely impact of interventions was evaluated using Kirkpatrick’s Hierarchy [23,24] by two reviewers. This framework uses four levels: Level 1 (participation), Level 2a (attitudes and perceptions) and Level 2b (knowledge and skills), Level 3 (behavioural change), and Level 4a (organisation practice) and 4b (patient benefits) to evaluate the impact of medical education research. This model considers educational beneficiaries from the student, organisation, and patient perspective [24]. 

## 3. Results

**Figure 2:**
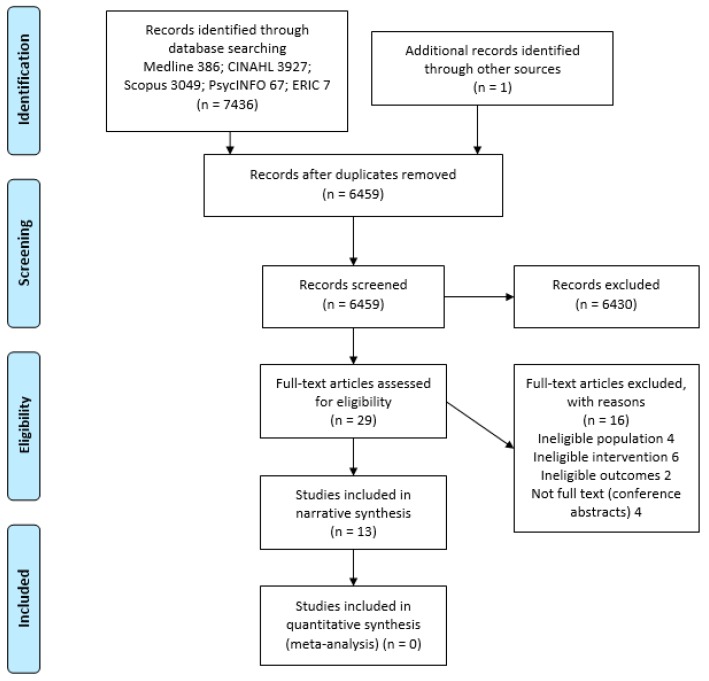
Study flow diagram.

Of 7436 studies identified through database searching (Figure 2) and one additional study located through hand-searching of the reference lists, the final library consisted of 13 studies (Table 1). Nine [25,26,27,28,29,30,31,32,33] were quantitative studies, one [34] was a mixed methods study, and three [35,36,37] were qualitative studies.

Table 1 reports the key study characteristics of included studies. Literature was identified from the United States (US) and Canada, Australia, United Kingdom (UK), and Japan [25,26,27,28,29,30,31,32,33,34,35,36,37]. Students usually were identified as being in the later years of nutrition and dietetics programmes. Six studies utilised authentic patients [26,29,30,31,34,37]; simulated patients and actors were utilised in the remaining studies. One study [31] compared education involving authentic and simulated patients on communication skills and skills promoting behaviour change.

Patients tended to play a passive role in student education activities, mainly as recipients of care. Only three studies involved patients in providing feedback on student performance [34,35,36], and another had patients rate their experience and the students’ counselling skills [29].

Table 2 reports student skill development, including communication skills and interview or counselling skills associated with the patient education experience. Improved confidence in clinical skills [29,37], self-reflection [33], and professionalism [29] were also reported. These skills were gained irrespective of whether the education was undertaken with real or simulated patients. Patient-related outcomes were reported less frequently. Students in the study of Gibson et al. [34] demonstrated that they could perform the technical skills of malnutrition screening, referring patients at risk of malnutrition to a dietitian. Another study that reported students counselling a group of overweight and obese patients [26] delivered weight loss outcomes. Patient-centredness arising from the education process was not described. 

In evaluating the impact of educational programmes using Kirkpatrick’s Hierarchy [23,24], eight of the 13 studies were rated as Level 2a, whereby they sought to modify student attitudes or perceptions. Four studies were rated as Level 3, Behavioural change, where they sought to apply and/or evaluate new knowledge and skills of learners. Just one of the included studies [26] was rated as Level 4b, with benefits to the patient or client as a direct result of the learning intervention.

Table 3 reports the quality assessment of all studies. Of the qualitative and mixed methods studies, the report of Swanepoel et al. [37] rated highly across all aspects of the Critical Appraisal Skills Programme. Three studies [34,35,36] were downgraded for reasons including not adequately considering the relationship between researcher and study participants (both students and patients). Results ranged from 9 [25,30,33] to 13 [27] of a maximum 18 for quantitative studies. Downgrading occurred across the study library for studies conducted within only one sampling institution and for the use of non-validated outcome assessment tools. Two studies reported on randomised controlled trials [25,27]. 

## 4. Discussion

This review aimed to synthesise learning outcomes that result from involvement of patients in nutrition and dietetic student education, and to consider whether these interactions promote patient-centred care. Nutrition and dietetics education, consistent with broader healthcare curriculum, has a focus on delivering client-centred care [8]. However, given the number of studies identified for inclusion in this review, the translation of these concepts into an interactive student educational experience has been investigated to a somewhat limited extent. Outcomes for students from the participation of patients in their training were similar to those in the broader healthcare literature. Patient involvement in the education of dietitians supports skill development and therefore progression to competence. Development of skills and confidence, as well as placing learning into context, were identified as have been reported in previous reviews [38]. However, benefits to patients, such as creating a sense of empowerment or using their knowledge and experience of their condition, were not described [38]. 

The research outcomes identified in this review suggest that there has been limited evaluation of active patient involvement in the education of student dietitians/nutritionists. There were few measured benefits to patients receiving nutrition and dietetic student intervention, and none reported adverse effects. In addition, few reports were of patients providing feedback to students, with evaluations and feedback led predominantly by educators. Patient-reported outcomes and their perceptions of their own care, as compared to objective patient-based outcomes, are also valuable when assessing clinical education [39]. 

The patient voice—surely at the centre of patient-centred care—is not being heard across the body of research examined in this review. Patients may prioritise interpersonal abilities over clinical skills when evaluating their own health care [40], yet students typically receive feedback only from supervisors, peers, and occasionally staff from other disciplines. Although patient feedback does not always align with supervisor-assessed competence [40], excluding the patient voice in student education eliminates a vital and powerful source of feedback. 

The broader medical education literature describes demarcation between “authentic” patients, i.e., people who have direct lived experience with a particular illness or condition [15], and people who role-play as patients with conditions that they do not actually have (simulated patients). This review identified that in nutrition and dietetics, educational benefits can be gained from both simulated and authentic patients. While their educational roles differ from those of real patients able to contribute authenticity to curricular decisions [15], simulated patients can play a vital role in supporting student learning [41]. Other reports within healthcare education describe psychological stress for patients due to repeatedly describing their illness [42], as well as anxiety associated with being in a teaching role [43]. These issues pose challenges for future curriculum design and educational research, whereby power dynamics versus delivery of educational benefit should be considered and explored. 

Patients are unwell and vulnerable; within the environment of clinical education, they may also be considered exposed, tired, and frightened [3], and so their participation in teaching and learning should not be assumed. Although ethics committee approvals were obtained for included studies, ethical considerations of bedside learning were not reported in any of the included studies. These have been described more broadly within the medical literature but warrant consideration for nutrition and dietetic student education. Issues relating to obtaining patient consent of patient participation in student education and ethical considerations in trying to balance the healthcare needs of patients versus maximising learning opportunities for students [15] were not described. The rationale for choosing simulated patients over bedside learning was not considered in the studies included within this review, although cost effectiveness [44] may influence decision making.

This review had several strengths, including a broad search across five databases with no limits on language, time period, or outcomes to ensure that all relevant literature was included. A further strength was the broad scope of setting (beyond the traditional clinical setting) using search terms including client and consumer. This enabled students across different domains of practice in nutrition and dietetics professions to be included. 

The use of Kirkpatrick’s Hierarchy used within this review to evaluate the impact of medical education research has been acknowledged elsewhere for its limitations [24]. These limitations include that the model does not consider broader outcomes that may arise from different research methods. However, within this review, this hierarchy did highlight that limited benefits to patients of nutrition and dietetic student interactions have been measured to this point. Challenges in assessing whether interactions between patients and students actually promote patient-centred care are also acknowledged. As identified in the quality assessment, included studies were not all methodologically strong, which limits the outcomes that can be drawn. Although five databases were searched, as in all systematic reviews, there is a possibility that eligible papers have been missed. 

Given the limited description of active patient involvement in student education, there are many opportunities for future research. This research should be conducted and reported with transparent decision making and should describe why (or why not) different student and patient outcomes have been considered. Patients have the potential to provide valuable input and feedback beyond that which might be considered by educators, supervisors, peers, and indeed experienced practitioners. If student dietitians are to develop skills that service users perceive as worthwhile, their voice needs to be heard. The engagement with patients (including their consent, preparation, and training) to take on their educational role, whether they are authentic or simulated, should also be considered and reported. There is also opportunity for patients to contribute to the design of student learning through patient-centred educational research. Real engagement with patients with feedback on their experience through their participation in the curriculum is encouraged.

## 5. Conclusions

Education of nutrition and dietetics students should not exist now or into the future without the service end users. Transformation is needed—to engage and involve patients and other service users in nutrition and dietetics education. This is a challenge for our profession, where services and research involving patients and students can deliver the long-held aim of “nothing about me without me”. 

## Figures and Tables

**Table 1:** Study characteristics of included studies.

First Author Date, Country	Setting	Student Characteristics	Patient Characteristics	Study Design	Outcome Measures
Beshgetoor [25] 2007, USA	Simulated settings including clinics, hospital	Dietetic students (number unspecified) enrolled in a senior level community nutrition course	SP as patients of various cultural and nutritional backgrounds.	Post-test survey completed by students	Student perceived effectiveness and value
Brightwell [26] 1976, USA	Not specified	*n* = 10 undergraduate dietetic students	11 female and 1 male patients underwent weight reduction counselling by students. 9 patients (75% retention) completed the program	Mixed methods – patient anthropometry measurements and qualitative interviews of students	Post-programme patient weight change and student perceptions of counselling patients
Dobson [27] 2007, Canada	Osteoporosis assessment laboratory	pharmacy, nutrition and physical therapy *n* = 25 3rd year nutrition students	6 female SP role-played a patient with a newly diagnosed vertebral compression fracture	Pre- and post-test of researcher developed survey (6 statements, 7-point Likert scale) administered to students	Attitudes and experiences of working in inter professional team
Gibson [28] 2015, Australia	University	*n* = 215 3rd year undergraduate dietetic students	SP role-played a simple nutritional condition congruent with the concurrently taught academic content including communication skill development	Observational study using researcher-developed survey measuring communication skills by dietetic educators	Communication skill development
Gibson [34] 2016, Australia	Hospital	*n* = 58 pre-clinical 3rd year dietetic students	Hospital patients in medical, rehabilitation, surgical, and coronary care wards	Mixed method case-control study, using a survey developed by other authors and 6 focus groups at placement completion. Intervention group completed patient malnutrition screening activity; control group had usual orientation activities.	Student feelings of preparedness for placement; student perceptions of participating in screening activity
Hampl [35] 1999, USA	University	*n* = 14 undergraduate student dietitians	SP in role of 17-year-old pregnant woman	Qualitative evaluation of student-SP interviews using a questionnaire, primarily composed of open-ended questions	Student perceptions of participating in activity
Henry [36] 2009, USA	Family counselling centre	*n* = 11 dietetic interns (mean age 25 years) near midpoint of supervised practice experience.	4 SPs (mean age 25 years) recruited from the first-year graduate students in the marriage and family therapy programme.	Qualitative study using focus groups (*n* = 10 dietetic interns)	Student perceptions of effectiveness of SP sessions on counselling competence
Horacek [25] 2007, USA	Unspecified private location	*n* = 121 dietetic students and interns	Patients were identified through campus recreation services or a wellness programme.	Mixed methods: Students and supervising dietitians used a modified version of the Dietitian’s Interviewing Rating Scale to evaluate session. Clients evaluated the counselling experience and skills in a post-interview survey.	Student, supervising dietitian, and client perceptions of counselling sessions; outcomes were compared between groups.
Kim [30] 2003, USA	Senior Citizens Centre	*n* = 49 dietetic students enrolled in Nutrition for the Ageing unit	150 elderly people attending the Senior Citizens Centre for meals	Pre and post evaluation questionnaires	Student attitudes towards community service before and after service learning experiences
Schwartz [31] 2014,USA	University	*n* = 75 dietetic students	Real patients who were recruited from community for healthy eating or weight loss advice; SPs were actors with abdominal obesity	Retrospective evaluation of student-patient consultation video recordings (*n* = 138) by recent nutrition course graduates trained to use rating tool	Rating of student communication and promoting behaviour change skills
Simper [32] 2017, UK	Not specified	*n* = 52 students from a final year undergraduate nutrition cohort	Actor familiar with motivational interviewing approach	Repeated measures design where students were videoed and videos were coded for motivational interviewing behaviours	Student behaviour and global ratings related to motivational interviewing
Swanepoel [37] 2016, Australia	University weight management clinic	*n* = 13 third-year dietetic students (43% of total enrolment)	Clients were university staff attending the university weight management clinic	Qualitative study design: student focus groups used to explore the impact of participation	Student perceived confidence and skills
Tada [33] 2018, Japan	Private Japanese university	*n* = 90 third-year dietetics undergraduates aged 20–38 years	SPs as elderly patients in hospital and home setting	Pre- and post- survey using researcher-developed questionnaire	Student self-efficacy across 7 nutrition professional practice competencies

OSCE, objective structured clinical examination; SP, simulated patient; UK, United Kingdom; USA, United States of America.

**Table 2:** Study outcomes and Kirkpatrick’s Hierarchy of included studies.

First Author	Educational Role of the Patient	Student Related Outcomes (e.g., Learning, Perceptions)	Kirkpatrick’s Levels	Patient Related Outcomes
Beshgetoor [25]	SPs played patients with body weight issues, health-related risks, and resources for food procurement. Scenarios represented patients of diverse ages and ethnic backgrounds	Students perceived learning to be effective and the SP encounter useful for learning.	2a	None
Brightwell [26]	Overweight/ obese patients received weight reduction counselling by students	Learned new techniques; chance to work with real patients (and see them be successful); improved relationships with patients.	4b	All patients lost weight (2.0–18.2kg)
Dobson [27]	SP were interviewed for data collection	Students perceived they performed better than expected with regard to their ability to contribute to the interview, the patient care plan, and communication skills.	2a	None
Gibson [28]	SP provided feedback on student communication skills	No significant improvement in communication skills from formative to summative evaluation, but significant improvement made for failing and borderline students.	3	None
Gibson [34]	Hospital patients were screened for malnutrition	Improved student-perceived communication skills and mean of all skills (background knowledge, professional attributes, professionalism, communication skills, overall placement preparedness) in patient screening group compared with control group.	2a	Malnutrition screening performed satisfactorily
Hampl [35]	SP was interviewed and counselled by students and provided them with feedback	Students perceived the learning experience as valuable learning experience; range of perceptions regarding authenticity of setting, immediate feedback from SP, and instructor enhanced learning, SP feedback was helpful, informative, and encouraging.	2a	None
Henry [36]	SP provided feedback to students, however, it was unclear if this was in their patient capacity	Students reported varying levels of confidence, with videoing of session adding to anxiety; students perceived improved confidence and competence; valued opportunity to practise a second time.	2a	None
Horacek [29]	Real patients received dietetic counselling from students and rated the experience and students’ counselling skills	Improved skills in knowledge, preparedness, communication skills, confidence, flexibility, and professionalism.	3	High satisfaction of counselling experience
Kim [30]	Senior citizens received meal-related services and nutrition education by students	Improved knowledge of community; improved understanding of resources, health care needs, barriers to receiving health care, impact of SES on health, importance of community programmes. Developed communication and “people” skills, improved writing and presentation skills, better understanding of older people.	2a	None
Schwartz [31]	Real patients and SPs were counselled for weight reduction where the session was videoed	Quality of communication and behaviour change counselling skills were assessed as good to excellent, however change in student learning as a result of the intervention was not measured.	3	None
Simper [32]	SP received motivational interviewing from students	Improved motivational interviewing skills including reduced closed questions, increased reflections and affirmations, and reduced student: client speaking time ratio.	3	None
Swanepoel [37]	University staff attending a university-based weight management clinic were interviewed by students	Increased professional confidence; increased confidence in clinical skills; improved students’ perceived ability to identify skills required for practice; developed sense of professional identity; feedback from supervisor.	2a	None
Tada [33]	SP played role of an elderly person in hospital and home settings	Improved self-efficacy in ethics, interpersonal skills, nutrition assessment, diagnosis, and care planning skills. Students reported enhanced learning and understanding, self-reflection, and confidence.	2a	None

SP, simulated patients; UK, United Kingdom; USA, United States of America; Kirkpatrick’s Levels [17]: 2a = attitudes and perceptions, 3 = behavioural change, 4b = patient benefits.

**Table 3:** Quality assessment of included studies.

Author(s)	Q1	Q2	Q3	Q4	Q5	Q6	Q7	Q8	Q9	Q10	Total
**Qualitative Studies Assessed Using the Critical Appraisal Skills Programme^1^**											
Gibson [28]	Y	Y	Y	Y	Y	N	Y	Y	Y	Y	N/A
Hampyl [35]	N	Y	N	Y	N	N	N	N	N	N	N/A
Henry [36]	Y	Y	Y	N	Y	N	N	Y	Y	Y	N/A
Swanepoel [37]	Y	Y	Y	Y	Y	Y	Y	Y	Y	Y	N/A
**Quantitative and mixed method studies assessed using the Medical Education Research Study Quality Instrument^2^**											
Beshgetoor et al. [25]	3	0.5	0.5	1	0	0	0	1	2	1	9
Brightwell [26]	1.5	0.5	1.5	3	0	0	0	1	1	3	11.5
Dobson et al. [27]	3	0.5	1.5	3	0	0	0	1	2	2	13
Gibson & Davidson [34]	1.5	0.5	1.5	3	0	0	0	1	2	2	11.5
Horacek et al. [29]	1.5	0.5	1.5	3	0	0	0	1	2	1.5	11
Kim et al. [30]	1.5	0.5	1.5	1	0	0	0	1	2	1.5	9
Schwartz et al. [31]	1	0.5	1.5	3	0	0	0	1	2	1.5	10.5
Simper [32]	1.5	0.5	1	3	0	0	0	1	2	2	11
Tada [33]	1.5	0.5	1.5	1	0	0	0	1	2	1.5	9

^1^ determined using the Critical Appraisal Skills Programme where: Q1 Was there a clear statement of the aims of the research?; Q2 Is a qualitative methodology appropriate?; Q3 Was the research design appropriate to address the aims of the research?; Q4 Was the recruitment strategy appropriate to the aims of the research?; Q5 Was the data collected in a way that addressed the research issue?; Q6 Has the relationship between researcher and participants been adequately considered?; Q7 Have ethical issues been taken into consideration?; Q8 Was the data analysis sufficiently rigorous?; Q9 Is there a clear statement of findings?; Q10 How valuable is the research?. Response options to all questions were: Y Yes; C Can’t tell; N No. ^2^ determined using the Medical Education Research Study Quality Instrument where: Q1 Study design (total score 3); Q2 Sampling institutions (1.5); Q3 Sampling response rate (1.5); Q4 Type of data (3); Q5–7 Validation of evaluation instrument (3); Q8–9 Data analysis (3); Q10 Outcomes (3); Total (18).
